# Degradation Mechanism of 2,4-Dichlorophenol by Fungi Isolated from Marine Invertebrates

**DOI:** 10.3390/ijms21093317

**Published:** 2020-05-07

**Authors:** Efstratios Nikolaivits, Andreas Agrafiotis, Eirini Baira, Géraldine Le Goff, Nikolaos Tsafantakis, Suchana A. Chavanich, Yehuda Benayahu, Jamal Ouazzani, Nikolas Fokialakis, Evangelos Topakas

**Affiliations:** 1 Industrial Biotechnology & Biocatalysis Group, Biotechnology Laboratory, School of Chemical Engineering, National Technical University of Athens, 15780 Athens, Greece; snikolai@central.ntua.gr (E.N.); agrafiotisandreas.vep@gmail.com (A.A.); 2 Division of Pharmacognosy and Chemistry of Natural Products, Department of Pharmacy, National and Kapodistrian University of Athens, 15771 Athens, Greece; ebaira@pharm.uoa.gr (E.B.); ntsafantakis@pharm.uoa.gr (N.T.); fokialakis@pharm.uoa.gr (N.F.); 3 Institut de Chimie des Substances Naturelles, ICSN, CNRS, 91198 Gif sur Yvette, France; geraldine.legoff@cnrs.fr (G.L.G.); jamal.ouazzani@cnrs.fr (J.O.); 4 Faculty of Science, Chulalongkorn University, Bangkok 10330, Thailand; suchana.c@chula.ac.th; 5 School of Zoology, Faculty of Life Sciences, Tel Aviv University, Tel Aviv 69978, Israel; YehudaB@tauex.tau.ac.il; 6 Biochemical and Chemical Process Engineering, Division of Sustainable Process Engineering, Department of Civil, Environmental and Natural Resources Engineering, Luleå University of Technology, SE-97187 Luleå, Sweden

**Keywords:** 2,4-dichlorophenol, marine-derived fungi, invertebrate symbionts, catechol dioxygenase, DCP metabolites

## Abstract

2,4-Dichlorophenol (2,4-DCP) is a ubiquitous environmental pollutant categorized as a priority pollutant by the United States (US) Environmental Protection Agency, posing adverse health effects on humans and wildlife. Bioremediation is proposed as an eco-friendly, cost-effective alternative to traditional physicochemical remediation techniques. In the present study, fungal strains were isolated from marine invertebrates and tested for their ability to biotransform 2,4-DCP at a concentration of 1 mM. The most competent strains were studied further for the expression of catechol dioxygenase activities and the produced metabolites. One strain, identified as *Tritirachium* sp., expressed high levels of extracellular catechol 1,2-dioxygenase activity. The same strain also produced a dechlorinated cleavage product of the starting compound, indicating the assimilation of the xenobiotic by the fungus. This work also enriches the knowledge about the mechanisms employed by marine-derived fungi in order to defend themselves against chlorinated xenobiotics.

## 1. Introduction

The progress of industrialization and increase in various human activities increased the use of various chemicals in various consumer products, drugs, pesticides, food additives, fuels, and industrial solvents. Pollution of air, water, and soil can occur as a result of the improper disposal of said chemicals [1]. There is recently heightened concern among policymakers and scientists with regard to the effects of human and wildlife exposure to chemical compounds in the environment, particularly the aquatic environment [2]. Application of pesticides, insecticides, and herbicides constitutes the main source of water pollution with phenolic compounds through an agricultural source.

The treatment of pollutants performed via conventional methods (both physical and chemical) is a costly, time-consuming approach that generates other contaminants in the process [3]. According to an assessment, restoring of contaminated sites in the United States of America (USA) requires a capital investment of ca. $1.7 trillion United States dollars (USD). Hence, bioremediation emerged as a safe, low-cost, and environmentally friendly alternative technology to achieve sustainable removal of hazardous pollutants [1]. The US Environmental Protection Agency (USEPA) defines bioremediation as a treatment process in which microorganisms are employed to degrade or modify toxic pollutants to less harmful products, thus reducing environmental pollutants generated by various anthropogenic activities.

Phenolic pollutants are recalcitrant to biodegradation, due to their aromatic structure that is hard to cleave. The first step toward the degradation of such pollutants is usually their hydroxylation performed by monooxygenases [4], leading to catecholic derivatives (catechol, protocatechuate, or hydroxyquinol), which are subsequently transformed by ring-cleaving dioxygenases. The cleavage of the ring is a very important step toward the mineralization of the molecule and is performed by inserting two oxygen atoms of molecular oxygen either between (*ortho* cleavage) or beside (*meta* cleavage) the two hydroxyl groups of the ring [5]. Intradiol dioxygenases (*ortho* pathway) are Fe^3+^-containing enzymes, while extradiol dioxygenases (*meta* pathway) contain an Fe^2+^ center for catalysis [6]. Extradiol dioxygenases also include the cupin-type dioxygenases, which act on noncatecholic hydroxy-substituted aromatic carboxylic acids (gentisate, salicylate, 1-hydroxy-2-naphthoate, or aminohydroxybenzoates) [7].

The marine environment is an untapped source of microbial diversity, showing various characteristics valuable for biotechnological applications, including bioremediation [8]. Marine and estuarine environments are repositories of numerous organic pollutants, such as petroleum hydrocarbons, chlorinated and brominated organics, plastics, etc. The halogenated organics have natural sources; thus, marine environments naturally harbor a great microbial diversity for the degradation of those types of organic compounds, which are involved in a microbial degradation network [9]. Fungi are robust organisms, and most of them are usually more tolerant to high concentrations of pollutants compared to bacteria; hence, they are more efficient in bioremediation compared to other microorganisms [10].

The present work describes the screening of 27 fungal strains isolated from three different marine ecosystems as symbionts of different invertebrates (such as sponges, corals, tunicates, and bivalves) from the east Mediterranean Sea, Red Sea, and Andaman Sea. In order to enrich the catalog of biocatalysts able to convert chlorinated aromatic pollutants, these strains were screened for their degradation potential of 2,4-dichlorophenol (2,4-DCP), which was considered as a model di-halogenated aromatic compound.

## 2. Results and Discussion

### 2.1. Biotransformation Potential of 2,4-DCP by Marine-Derived Fungi

In our previous work [11], we studied the bioremediation potential of symbiotic fungal strains deriving from different depths (30–152 m) of the mesophotic marine zone (upper, middle, and lower). In the present work, invertebrates collected from the east Mediterranean Sea and the Red Sea were found in the upper mesophotic zone (35–49 m), while those from the Andaman Sea were found from shallow reefs (6–15 m). Additionally, the collection of invertebrates took place in different time periods in each case. The microorganisms were isolated as invertebrate symbionts and cultivated in marine broth, in order to maintain the salinity of the medium. The non-mesophotic isolates were dominated by *Penicillium* (54%) and *Aspergillus* (31%) species. Mesophotic strains were more diverse, being represented mostly by *Aspergillus* (29%), *Penicillium* (21%), and *Aphanoascus* (21%) species.

Most of the 27 isolates screened showed significant 2,4-DCP bioconversion potential. The decrease in 2,4-DCP concentration at the end of the resting-cell incubation (10 days) is presented in Table 1. The overall average removal yield was 33%, and it was the same for the mesophotic and non-mesophotic strains. The majority of strains (74%) could transform 2,4-DCP at yields between 20% and 50%. In our previous study [11], again, the majority of marine-derived strains reached removal yields between 20% and 50%, and the overall average was similar (32%). Out of the 27 strains in this study, only four were able to reach bioconversion yields over 55%, namely, *Cladosporium* sp. ML6-S1 (64.0%), *Aspergillus* sp. ML147-S2 (55.1%), *Penicillium chrysogenum* ML156-S8 (59.5%), and *Tritirachium* sp. ML197-S3 (66.3%). These strains seem to be different from those studied in our previous work, such as *Penicillium steckii*, *Chrysosporium* sp., *Penicillium* sp., *Aspergillus creber*, and *Aspergillus* sp. The initial concentration of 2,4-DCP used (163 mg∙mL^−1^) is higher than most reported so far. Concentrations of 40–100 mg∙mL^−1^ were tested by various researchers, and the removal yields were between 20% and 65% [12,13,14,15].

### 2.2. Expression of Catechol Dioxygenase Activities

**Figure 1:**
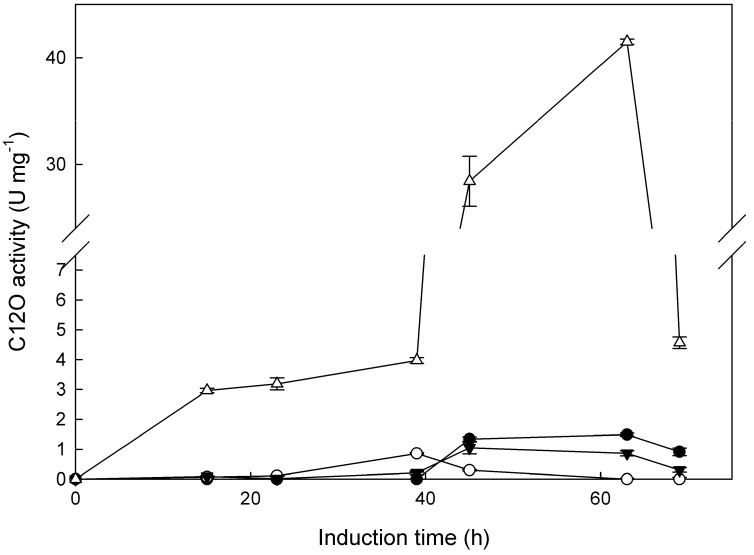
Time-course of the extracellular catechol 1,2-dioxygenase (C12O) activity expressed as U∙mg^−1^ of protein for the four selected strains, induced by 1 mM 2,4-DCP. Strains: *Cladosporium* sp. ML6-S1 (●), *Aspergillus* sp. ML147-S2 (○), *Penicillium chrysogenum* ML156-S8 (▼), and *Tritirachium* sp. ML197-S3 (∆).

Extracellular catechol dioxygenase activities were measured in the culture broth of the tested strains following induction with 2,4-DCP. All tested strains seemed to mostly express catechol 1,2-dioxygenase (C12O) instead of catechol 2,3-dioxygenase (C23O) activity. C23O activity is expressed in very low levels, and just two strains—*Tritirachium* sp. ML197-S3 and *Aspergillus* sp. ML147-S2—presented detectable activity at 63 h of 0.36 and 0.33 U∙mg^−1^, respectively. As seen in Figure 1, the majority of strains, except for *Aspergillus* sp. ML147-S2, presented the maximum C12O activity at 63 h. Additionally, it is clear that the strain *Tritirachium* sp. ML197-S3 had the ability to express high C12O activity (41.50 U∙mg^−1^) compared to the rest of the tested strains, being 28-fold higher than the second highest strain (*Cladosporium* sp. ML6-S1). Intracellular C12O activity of the strain *Tritirachium* sp. ML197-S3 was also measured at the peak point of extracellular activity. While the activity detected in the extracellular medium was found to be 0.55 U, the intracellular one was 0.13 U, which constitutes only 19% of the total C12O activity detected. *Aspergillus awamori* NRRL 3112, which had the ability to grow on several phenolic compounds, expressed 0.043 U∙mg^−1^ of extracellular C12O when grown on 2,4-DCP [16]. The same strain, when grown on phenol and catechol, expressed higher specific C12O activity both intra- and extracellularly.

Bacterial intradiol dioxygenases were extensively investigated regarding their reaction mechanism, substrate specificity, and structures [5]. C12O enzymes are known to be expressed by various microorganisms able to degrade phenolic pollutants. Bacteria able to assimilate 3-hydroxybenzoate [17], phenol [18], benzoic acid [19], and α-naphthol [20] express such enzymes that were characterized. On the contrary, the reports about C12O expression by fungi, mostly related to their biochemical characterization, are scarce. Phenol-induced C12O activity was detected in various filamentous fungi [21,22], and only a few reports on isolation and characterization from *Candida* strains were published [23,24].

### 2.3. Identification of 2,4-DCP Metabolites

The bacterial mechanisms for the detoxification and assimilation of chlorophenols are well known and were reviewed recently [25]. On the other hand, the studies regarding the defensive mechanisms of fungi for the handling of chlorophenols and especially 2,4-DCP are limited. In general, fungi are known to utilize a two-step process for the detoxification of xenobiotics. During phase I, they express enzymes, typically belonging to the cytochrome P450 family, which modify the initial compound by adding functional groups, such as –OH. The resulting compounds are then further modified by phase II enzymes that include several non-specific transferases (sulfo-, glycosyl-, glutathione-, etc.). Their products are less toxic and are excreted from the cells without any further modification [26,27].

Based on the MS analysis, several compounds were detected. However, the emphasis was given to compounds related to the 2,4-DCP metabolism study. For the annotation of 2,4-DCP metabolites, various software tools and online databases were employed. For the prediction of the elemental composition (EC) of the compounds, software tools such as Sirius, Rdisop, and mMass were employed according to the accurate mass, the relative intensities of the first and second isotopes of each compound, the composition rules of H/C, NOPS/C, and Ring Double Bond Equivalent value (RDBE), and applying an *m/z* tolerance of 10 ppm. The online databases Metlin and Chemspider, as well as data from literature, were used to annotate the metabolites with an applied *m/z* tolerance of 10 ppm. All the compounds that are characterized as metabolites were only detected on DCP-treated cell cultures. Table 2 depicts the 2,4-DCP metabolites based on the analysis.

Nine metabolites of 2,4 DCP **(10)** were identified, where some are still holding a chlorine substituent. For *Aspergillus* sp. ML147-S2 and *Tritirachium* sp. ML197-S3, six metabolites were detected, while, for the remaining two strains, only four metabolites were detected. All of the tested strains were able to hydroxylate 2,4-DCP to generate an *ortho* diol **(9)**, which is appropriate for further dioxygenase reactivity [25,28]. All of the selected strains are able to substitute the two chlorines of 2,4-DCP by hydroxyl groups leading to trihydroxybenzene (or hydroxyquinol) **(5)**. All strains except for *Aspergillus* sp. ML147-S2 were able to transform compound **(9)** into tetrahydroxybenzene **(4)**. The aforementioned reactions are performed by phase I enzymes mostly cytochrome-P450 monooxygenases. This intense dechlorination capacity of the selected strains revealed a specific detoxification activity proportionally related to the number of chlorines on the aromatic ring. The further glycosylation of **(9)** to **(8)** by *Aspergillus* sp. ML147-S2 and *Tritirachium* sp. ML197-S3 is aimed at enhancing compound removal from the cells as the final step of detoxification. Finally, except for *Cladosporium* sp. ML6-S1, all the strains were able to convert compound **(9)** to the corresponding glutamine conjugate **(3)**. Complete dechlorination of the starting xenobiotic is really important, since it substantially decreases its toxicity. However, there was also a non-dechlorinated product of dichlorocatechol in a glycosylated form **(8)**. This metabolite was found in strains *Aspergillus* sp. ML147-S2 and *Tritirachium* sp. ML197-S3, and, even though it is much less lipophilic than its precursor, it still contains both chlorine atoms and it is probably a dead-end product. Dichlorocatechol could also be partially dechlorinated by all strains, except for *Cladosporium* sp. ML6-S1, to form the respective glutamine conjugate **(3)**.

Other partially dechlorinated products were also detected, such as chlorophenol **(1)** in *Aspergillus* sp. ML147-S2 and sulfated **(7)** and cysteine **(6)** conjugates in *Aspergillus* sp. ML147-S2 and *Cladosporium* sp. ML6-S1.

In the reaction of *Tritirachium* sp. ML197-S3, based on HRMS data, 2-hydroxymuconic acid **(2)** was detected. This compound may derive from a dioxygenase catalyzed cleavage of **(5)**. This could be correlated to the high C12O activity detected in this strain. This is a very significant finding, since there is a high probability of 2,4-DCP being assimilated by this particular strain. However, this result is in contrast to the degradation mechanism of 2,4-DCP by bacteria, where the cleavage of the ring is performed before the dehalogenation of the compound [29].

### 2.4. Metabolic Pathways of 2,4-DCP

As previously reported with exclusively mesophotic fungi [11], dioxygenase activities were found in addition to no cleavage products. However, hydroxyquinol was detected, which was the only fully dechlorinated metabolite. Despite that, the metabolites of mesophotic fungi were more diverse compared to the fungi in the present study. However, several compounds were detected in both cases, such as dichlorocatechol, its glycoside, and its glutamine conjugate, as well as chlorophenol and its sulfated and cysteine conjugates. The isolated *Aspergillus* strains from the two studies, *Aspergillus* sp. ML147-S2, *Aspergillus creber* TM122-S3, and *Aspergillus* sp. TM124-S1, had only two metabolites in common: sulfated chlorophenol and the glutamine conjugate of chlorocatechol. On the other hand, *Penicillium chrysogenum* ML156-S8 had only one common metabolite with *Penicillium* sp. TM38-S1 [11].

Other than that, little is known about the 2,4-DCP metabolites of other fungi. Soil fungus *Mortierella* sp. used two different pathways for the detoxification of 2,4-DCP. The first included its hydroxylation to dichlorocatechol similar to the present study and its further methylation to dichloroguaiacol, while the second involved its oxidative dechlorination to 2-chloro-hydroquinone and its further reductive dehalogenation to hydroquinone [28]. Some of the metabolites of 2,4-DCP by white-rot fungus *Phanerochaete chrysosporium* were also identified to be 2,4-dichloroanisole, 2-chloro-1,4-hydroquinone, and 2-chloro-1,4-dimethoxybenzene [30]. *P. chrysosporium* was also able to completely dechlorinate the starting compound, yielding 2,5-dimethoxy-1,4-hydroquinone and 1,2,4,5-tetrahydroxybenzene.

**Figure 2:**
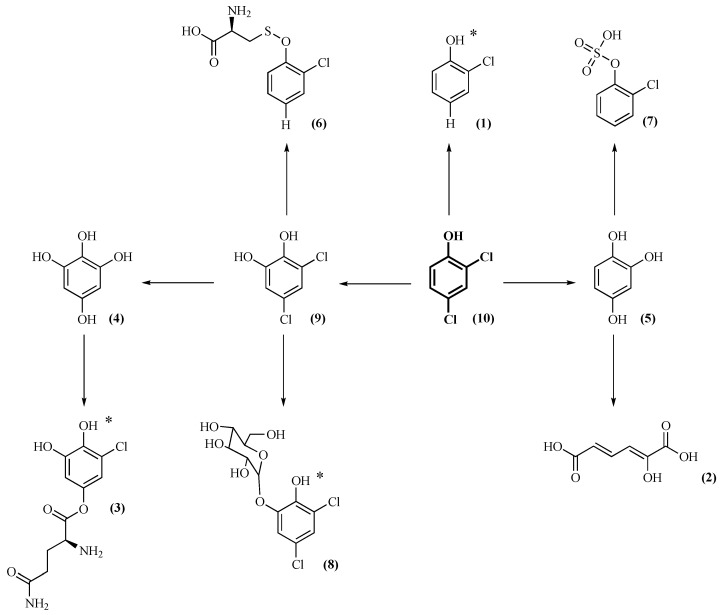
Proposed metabolic pathway for the detoxification of 2,4-DCP by the isolated fungi. The isomers were suggested according to MS and literature data. For the metabolites where there is no information about the most probable isomer, an asterisk was added next to the molecule. The number next to each compound is that corresponding to Table 2.

Based on literature data and MS results presented in Table 2, the metabolic pathway utilized by the investigated fungal strains for the detoxification of 2,4-DCP was envisaged. Structural configurations presented in Figure 2 are tentative and are based on the most probable conformation according to the compound dynamics and data reported in literature.

The biotransformation yield of 2,4-DCP by the studied isolates cannot be directly correlated with the metabolites detected. Undoubtedly, catechol dioxygenase activities and the presence of a ring cleavage product are very important factors that probably enhance the overall biotransformation yield; however, they are not the only ones. As seen with the identified metabolites, different enzymes take part in the initial 2,4-DCP transformation. When other enzymes can act on these initial metabolites, then the first enzymes can presumably act even more on 2,4-DCP. What is also important is the formation of compounds that can be further processed by the microbial metabolism and not just dead-end products. Definitely, the overall biotransformation yield is not as crucial as the quality of the produced metabolites and, more specifically, their dichlorination process, since our main goal is the detoxification of the starting pollutant.

## 3. Materials and Methods

### 3.1. Chemicals

2,4-Dichlorophenol (99%) was purchased from Sigma-Aldrich (St. Louis, MO, USA). Organic solvents (acetonitrile and chloroform) were of HPLC grade (Fisher Chemical, Pittsburgh, PA, USA).

### 3.2. Isolation and Identification of Invertebrate Symbionts

After sampling, invertebrates were transferred carefully to the laboratory to be processed. Small pieces of tissue were taken from the samples before frozen. Invertebrate samples (1 cm^3^) were ground in sterile seawater and heated at 50 °C for 1 h. The suspension was serially diluted, plated on Difco™ Marine Broth Agar (BD Biosciences San Jose, CA, USA), and incubated at 28 °C for six weeks. A single colony was picked from the agar and cultivated as a pure culture on Difco™ Potato Dextrose Agar and Difco™ Marine Broth Agar (BD Biosciences San Jose, CA, USA) (media and cultivated for five days at 28 °C. The strain spores and mycelium were recovered by a gentle scratch of the agar plate surface using a scalpel, and they were conserved at −20 °C in 10% glycerol solution.

Genomic DNA of the purified strains was isolated using a DNeasy Plant Mini Kit (Qiagen, Hilden, Germany), according to the manufacturer’s instructions. The ITS region was amplified with primers ITS1F (5′–CTTGGTCATTTAGAGGAAGTAA–3′) and ITS4 (5′–TCCTCCGCTTATTGATATGC–3′) using described polymerase chain reaction (PCR) conditions. Amplicons were sequenced by Sanger sequencing (GATC, Eurofins Genomics, Ebersberg, Germany), and the sequences were aligned against the non-redundant database of NCBI using the BLASTn program.

### 3.3. Culture Conditions and Resting-Cell Reactions

The culture procedure followed was the same as previously reported [11]. Fungal strains were grown on Difco™ Marine Agar 2216 (BD Biosciences San Jose, CA, USA) plates containing 100 μg∙mL^−1^ Ampicillin at 27 °C for five days. Mycelia from these were used to inoculate submerged cultures with Difco™ Marine Broth 2216 (BD Biosciences San Jose, CA, USA) (pH 7.6) at 27 °C and 160 rpm. After five days, the biomass was filtered using 0.2-μm-pore Supor**^®^** polyethersulfone (PES) membrane disc filters (Pall Corporation, Port Washington, NY, USA) and used as a biocatalyst (10% *w*/*v*) in 15-mL reactions containing 1 mM 2,4-DCP in ultrapure water. Reactions with just 2,4-DCP were used as controls for the abiotic transformations. Furthermore, for each strain, control reactions with the same amount of biomass but no addition of 2,4-DCP were also realized. All reactions were left at 27 °C and 120 rpm for 10 days. Samples were withdrawn on the third and sixth days and analyzed after filtration. On the final day, the remaining reaction was extracted with equal volume of chloroform, and it was analyzed after drying and resolubilization in ultrapure water.

### 3.4. Detection and Quantification of 2,4-DCP

The quantification of 2,4-DCP was performed using the same method, as previously reported [11] using a SHIMADZU LC-20AD HPLC equipped with a SIL-20A autosampler (Kyoto, Japan). A C-18 reverse-phase NUCLEOSIL^®^ 100-5 (Macherey-Nagel, Dueren, Germany) served as the stationary phase and 40% aqueous acetonitrile served as the mobile phase at a flow rate of 0.8 mL∙min^−1^. Detection took place with the photodiode array detector Varian ProStar (Varian Inc., Palo Alto, CA, USA) at 285 nm. The total running time was 16 min and the retention time of 2,4-DCP was 12.4 min.

### 3.5. Identification of 2,4-DCP Metabolites by LC–MS

The analysis was performed on an ESI-LTQ-Orbitrap Discovery XL mass spectrometer (Thermo Scientific, San Jose, CA, USA) connected to an Accela UHPLC system (Thermo Scientific, San Jose, CA, USA). A Fortis UPLC C18 (2.1 × 100 mm, 1.7 μm) reverse-phase column (Fortis Technologies Ltd., Neston, UK) was used for the analysis. The mobile phase was a mixture of 0.1% (*v*/*v*) formic acid/water (solvent A) and acetonitrile (solvent B). Sample analysis was carried out in both positive (ESI+) and negative (ESI−) ion mode. The flow rate was 0.4 mL∙min^−1^. A gradient method of 30 min was used for the analysis as follows: 0 to 24 min: 95% A, 5% B; 24 to 28 min: 5% A, 95% B; 28 to 30 min: 95% A, 5% B. The column temperature was maintained at 40 °C and the injection volume was 10 μL. The conditions for the HRMS in each ionization mode were set as follows: for the positive ion mode, the capillary temperature and voltage were set at 320 °C and 40 V, respectively. The sheath gas flow was set to 40, and the aux gas flow was set to 8 arb units. The spray voltage was set to 3.6 kV, and the tube lens voltage was set to 120 V. For the negative ion mode, the capillary temperature and voltage were set to 320 °C and −20 V, respectively. The sheath gas flow was set to 40, and the aux gas flow was set to 8 arb units. The spray voltage was set to 2.7 kV, and tube lens voltage was set to −80 V. In both positive and negative ion mode, analysis was performed using the Fourier-transform mass spectrometer (FTMS) (Thermo Scientific, San Jose, CA, USA) full-scan ion mode. The Orbitrap resolution was set to 30,000 full width at half maximum (FWHM) and the data-dependent acquisition mode of the three most intense ions was used for studying the MS/MS fragmentation pattern in parallel to the acquisition of full-scan mass spectra. Data acquisition was performed for a mass range of 100–1000 Da, and the spectra were acquired in the centroid mode.

### 3.6. Measurement of Enzymatic Activities

Induction of enzymatic activities began by introduction of 1 mM 2,4-DCP (final concentration) in 50-mL fungal cultures that were left to grow for three days as mentioned above. Samples were withdrawn at frequent intervals and centrifuged at 14,000 × *g* for 10 min (10 °C) to remove the biomass. The supernatant was used as crude extracellular enzyme for the detection of catechol dioxygenase activities. After the last sample was taken, the remaining biomass was lysed in order to measure intracellular enzymatic activities. Lysis was initiated by adding 670 units of Lyticase (#L4025; Sigma-Aldrich, St. Louis, MO, USA) per gram of biomass in 0.1 M potassium phosphate buffer pH 7.5 and left to incubate at 30 °C for 1 h. Afterward, 10 mL of the same buffer with 1.2 M sorbitol and 0.5 mM MgCl_2_ was added to the reaction. The biomass suspension was sonicated at 4 °C in a VC505 Vibra-Cell Processor (Sonics & Materials Inc., Newtown, CT, USA) for four cycles of sonication (1 min of 8-s pulse followed by 8-s pause). The resulting suspension was centrifuged for 20 min at 20,000 × *g* (4 °C), and the resulting supernatant was used as intracellular crude enzyme for enzymatic assays.

A typical assay (250 μL final volume) contained 1 mM catechol as substrate in 50 mM Tris-HCl pH 7 buffer. The reaction began with the addition of 25 μL of crude enzyme and its time-course was recorded on a SpectraMax-250 microplate reader (Molecular Devices, Sunnyvale, CA, USA) equipped with SoftMaxPro software (version 1.1, Molecular Devices, Sunnyvale, CA, USA) set at 35 °C. One unit (U) of catechol 1,2-dioxygenase (C12O) activity is defined as the amount of enzyme that produces 1 μmol *cis*,*cis*-muconic acid per minute under the assay conditions. One unit (U) of catechol 2,3-dioxygenase (C23O) activity is defined as the amount of enzyme that produces 1 μmol 2-hydroxymuconic semialdehyde per minute under the assay conditions. The products of C12O and C23O were detected at 260 nm and 375 nm, respectively, and they were quantified according to Lin and Milase [18] and Hupert-Kocurek et al. [31].

## 4. Conclusions

The present work aimed towards the expansion of the biocatalytic toolbox for the bioremediation of chlorinated aromatic pollutants. Bioprospecting of novel microorganisms was achieved by accessing marine regions at various depths and collecting invertebrates. Fungal symbionts of these invertebrates were isolated, identified, and screened for their ability to transform high 2,4-DCP concentrations. The most competent strains were studied further in order to elucidate the mechanisms which they use in order to cope with this chlorinated pollutant. Since these strains originate from pristine habitats, their enzymatic arsenal is not evolved specifically for the biotransformation of this xenobiotic. In fact, these strains seemed to employ non-specific pathways, which nevertheless could lead to less toxic and, in some cases, fully dechlorinated metabolites. Surprisingly, one of the strains had the ability to cleave the aromatic structure of the pollutant following its dechlorination. This suggests the assimilation of the xenobiotic by this strain, which is the main objective of every bioremediation process. When tested for ring-cleavage activities, the same strain expressed the highest catechol dioxygenase activity, demonstrating the key enzyme for efficient bioremediation. 2,4-DCP can be considered as a model chlorinated aromatic pollutant, and strains with the ability to detoxify this compound are candidates for the bioremediation of other chlorinated xenobiotics. In our following studies, we will focus on the complete elucidation of the detoxification mechanism of chlorinated pollutants by these fungal strains using transcriptomic and genomic analyses [32].

## Figures and Tables

**Table 1:** Percentage of 2,4-dichlorophenol (2,4-DCP) removal in resting-cell reactions after 10 days for all isolated fungal strains, which were identified based on their internal transcribed spacer (ITS) sequence. Information (region and depth) about the invertebrate host of each strain is given. Locations: Red Sea (Red), east Mediterranean Sea (Med E), and Andaman Sea (Andaman).

Isolate	Invertebrate	Location	Depth (m)	Isolate Identification	% DCP Removal
ML119-S1	Bivalve (*Pteria aegyptiaca*)	Red	35	*Aphanoascus fulvescens*	25.4
ML123-S2	Sponge (on top of bivalve)	Red	35	*Cladosporium halotolerans*	21.1
ML132-S1	*Amphimedon ochracea*	Red	35	*Aspergillus fumigatus*	27.3
ML133-S2	Soft coral *(Sarcophyton glaucum)*	Red	35	*Cladosporium halotolerans*	9.3
ML136-S2	Bivalve (*Pteria aegyptiaca*)	Red	35	*Aspergillus* sp. (*fumigatus*)	41.4
ML147-S1	*Axinella verrucosa*	Med E	47	*Aspergillus terreus*	26.9
ML147-S2	*Axinella verrucosa*	Med E	47	*Aspergillus* sp. (*terreus*)	55.1
ML149-S1	*Sarcotragus muscarum*	Med E	49	*Aphanoascus fulvescens*	41.8
ML150-S1	*Sarcotragus muscarum*	Med E	45	*Aphanoascus fulvescens*	35.8
ML153-S1	*Sarcotragus muscarum*	Med E	45	*Penicillium fellutanum*	13.9
ML153-S2	*Sarcotragus muscarum*	Med E	45	*Penicillium fellutanum*	24.7
ML155-S1	*Sarcotragus muscarum*	Med E	35	*Lecanicillium* sp.	19.0
ML-156-S8	Ascidean	Med E	35	*Penicillium chrysogenum*	59.5
ML197-S3	*Protula intestinum*	Med E	6	*Tritirachium* sp.	66.3
ML6-S1	Sponge (*Chondrilla australiensis*)	Andaman	10–15	*Cladosporium* sp.	64.0
ML10-S1	Sponge (*Clathria (Thalysias) reinwardti*)	Andaman	10–15	*Penicillium coffeae*	37.1
ML14-S1	Sponge (*Phobas arborescens*)	Andaman	10–15	*Aspergillus niger*	22.9
ML15-S1	Sponge (*Phobas arborescens*)	Andaman	10–15	*Aspergillus terreus*	27.3
ML16-S2	Sponge (*Iotrochota baculifera*)	Andaman	10–15	*Penicillium chrysogenum*	37.9
ML45-S3	Hydroid (*Macrorhynchia philippina*)	Andaman	10–15	*Penicillium chrysogenum*	27.1
ML45-S5	Hydroid (*Macrorhynchia philippina*)	Andaman	10–15	*Penicillium steckii*	36.3
ML45-S6	Hydroid (*Macrorhynchia philippina*)	Andaman	10–15	*Purpureocillium lilacinum*	32.8
ML52-S1	Unknown hydroid	Andaman	10–15	*Penicillium citrinum*	26.7
ML52-S5	Unknown hydroid	Andaman	10–15	*Penicillium coffeae*	26.8
ML52-S6	Unknown hydroid	Andaman	10–15	*Aspergillus niger*	29.3
ML52-S7	Unknown hydroid	Andaman	10–15	*Penicillium steckii*	31.7
ML52-S8	Unknown hydroid	Andaman	10–15	*Aspergillus fumigatus*	32.8

**Table 2:** 2,4-DCP metabolites traced only in DCP-treated cell cultures. Rt = retention time; (M – H)^−^ = *m*/*z* of the pseudomolecular ion; EC = the elemental composition.

A/A	Rt (min)	(M – H)^−^	EC	Found In
(1)	0.89	126.9987	C_6_H_5_ClO	*Aspergillus* sp. MLm147-S2
(2)	1.11	157.0146	C_6_H_6_O_5_	*Tritirachium* sp. MLm197-S3
(3)	1.45	287.0449	C_11_H_13_O_5_N_2_Cl	*Tritirachium* sp. MLm197-S3
(3)	1.47	287.0452	C_11_H_13_O_5_N_2_Cl	*Tritirachium* sp. MLm197-S3, *Aspergillus* sp. MLm147-S2
(3)	1.50	287.0452	C_11_H_13_O_5_N_2_Cl	*P. chrysogenum* MLm156-S8
(4)	2.33	141.0197	C_6_H_6_O_4_	*Tritirachium* sp. MLm197-S3
(4)	2.44	141.0196	C_6_H_6_O_4_	*P. chrysogenum* MLm156-S8
(4)	2.45	141.0196	C_6_H_6_O_4_	*Cladosporium* sp. MLm6-S1
(4)	2.46	141.0196	C_6_H_6_O_4_	*Cladosporium* sp. MLm6-S1
(4)	2.50	141.0196	C_6_H_6_O_4_	*Cladosporium* sp. MLm6-S1
(5)	3.15	125.0248	C_6_H_6_O_3_	*Aspergillus* sp. MLm147-S2
(5)	3.18	125.0249	C_6_H_6_O_3_	*Aspergillus* sp. MLm147-S2
(5)	3.19	125.0248	C_6_H_6_O_3_	*Tritirachium* sp. MLm197-S3
(5)	3.19	125.0248	C_6_H_6_O_3_	*Cladosporium* sp. MLm6-S1
(5)	3.20	125.0247	C_6_H_6_O_3_	*Cladosporium* sp. MLm6-S1
(5)	3.20	125.0248	C_6_H_6_O_3_	*P. chrysogenum* MLm156-S8
(5)	3.22	125.0248	C_6_H_6_O_3_	*Tritirachium* sp. MLm197-S3
(5)	3.22	125.0248	C_6_H_6_O_3_	*Cladosporium* sp. MLm6-S1
(5)	3.23	125.0250	C_6_H_6_O_3_	*P. chrysogenum* MLm156-S8
(5)	3.25	125.0249	C_6_H_6_O_3_	*Aspergillus* sp. MLm147-S2
(5)	3.27	125.0250	C_6_H_6_O_3_	*Tritirachium* sp. MLm197-S3
(5)	3.27	125.0251	C_6_H_6_O_3_	*P. chrysogenum* MLm156-S8
(6)	6.93	245.9994	C_9_H_10_NClO3S	*Cladosporium* sp. MLm6-S1
(7)	7.34	206.9523	C_6_H_5_O_4_ClS	*Aspergillus* sp. MLm147-S2
(8)	10.62	339.0041	C_12_H_14_Cl_2_O_7_	*Tritirachium* sp. MLm197-S3
(8)	10.62	339.0042	C_12_H_14_Cl_2_O_7_	*Aspergillus* sp. MLm147-S2
(8)	10.64	339.0040	C_12_H_14_Cl_2_O_7_	*Tritirachium* sp. MLm197-S3
(8)	10.73	339.0041	C_12_H_14_Cl_2_O_7_	*Aspergillus* sp. MLm147-S2
(9)	11.91	176.9517	Cl_2_C_6_H_2_(OH)_2_	*Cladosporium* sp. MLm6-S1
(9)	11.92	176.9517	Cl_2_C_6_H_2_(OH)_2_	*Aspergillus* sp. MLm147-S2
(9)	11.93	176.9518	C_6_H_4_Cl_2_O_2_	*P. chrysogenum* MLm156-S8
(9)	11.94	176.9517	C_6_H_4_Cl_2_O_2_	*Tritirachium* sp. MLm197-S3
(9)	11.94	176.9517	Cl_2_C_6_H_2_(OH)_2_	*Cladosporium* sp. MLm6-S1
(9)	11.96	176.9521	C_6_H_4_Cl_2_O_2_	*P. chrysogenum* MLm156-S8
(9)	11.96	176.9521	C_6_H_4_Cl_2_O_2_	*Tritirachium* sp. MLm197-S3
(9)	12	176.9521	C_6_H_4_Cl_2_O_2_	*P. chrysogenum* MLm156-S8
(10)	13.46	160.9572	C_6_H_4_Cl_2_O	*Aspergillus* sp. MLm147-S2
(10)	13.46	160.9571	C_6_H_4_Cl_2_O	Control day 10
(10)	13.47	160.9571	C_6_H_4_Cl_2_O	*Tritirachium* sp. MLm197-S3
(10)	13.48	160.9572	C_6_H_4_Cl_2_O	*Aspergillus* sp. MLm147-S2
(10)	13.48	160.9572	C_6_H_4_Cl_2_O	*Cladosporium* sp. MLm6-S1
(10)	13.48	160.9572	C_6_H_4_Cl_2_O	*P. chrysogenum* MLm156-S8
(10)	13.49	160.9572	C_6_H_4_Cl_2_O	*Cladosporium* sp. MLm6-S1
(10)	13.50	160.9573	C_6_H_4_Cl_2_O	*P. chrysogenum* MLm156-S8
(10)	13.50	160.9571	C_6_H_4_Cl_2_O	*P. chrysogenum* MLm156-S8
(10)	13.51	160.9571	C_6_H_4_Cl_2_O	*Tritirachium* sp. MLm197-S3
(10)	13.51	160.9572	C_6_H_4_Cl_2_O	*Tritirachium* sp. MLm197-S3
(10)	13.52	160.9572	C_6_H_4_Cl_2_O	Control day 6
(10)	13.52	160.9573	C_6_H_4_Cl_2_O	*Aspergillus* sp. MLm147-S2
(10)	13.53	160.9572	C_6_H_4_Cl_2_O	*Cladosporium* sp. MLm6-S1
(10)	13.55	160.9572	C_6_H_4_Cl_2_O	Control day 2

## References

[1] Dhir B., Kumar R., Sharma A.K., Ahluwalia S.S. (2017). Bioremediation technologies for the removal of pollutants. Advances in Environmental Biotechnology.

[2] Kalogerakis N., Arff J., Banat I.M., Broch O.J., Daffonchio D., Edvardsen T., Eguiraun H., Giuliano L., Handå A., López-de-Ipiña K. (2015). The role of environmental biotechnology in exploring, exploiting, monitoring, preserving, protecting and decontaminating the marine environment. N. Biotechnol..

[3] Gul R., Kumar R., Kumar R., Anil Kumar S., Sarabjeet Singh A. (2017). Introduction to Environmental Biotechnology. Advances in Environmental Biotechnology.

[4] Arora P.K., Srivastava A., Singh V.P. (2010). Application of monooxygenases in dehalogenation, desulphurization, denitrification and hydroxylation of aromatic compounds. J. Bioremediat. Biodegrad..

[5] Guzik U., Hupert-Kocurek K., Wojcieszysk D., Chamy R., Rosenkranz F. (2013). Intradiol dioxygenases—The key enzymes in xenobiotics degradation. Biodegradation of Hazardous and Special Products.

[6] Siegbahn P.E.M., Haeffner F. (2004). Mechanism for catechol ring-cleavage by non-heme iron extradiol dioxygenases. J. Am. Chem. Soc..

[7] Fetzner S. (2012). Ring-cleaving dioxygenases with a cupin fold. Appl. Environ. Microbiol..

[8] Nikolaivits E., Dimarogona M., Fokialakis N., Topakas E. (2017). Marine-derived biocatalysts: Importance, accessing and application in aromatic pollutant bioremediation. Front. Microbiol..

[9] Atashgahi S., Häggblom M.M., Smidt H. (2018). Organohalide respiration in pristine environments: Implications for the natural halogen cycle. Environ. Microbiol..

[10] Kumari M., Ghosh P., Thakur I.S., Varjani S., Agarwal A., Gnansounou E.G.B. (2018). Application of microbes in remediation of hazardous wastes: A review. Bioremediation: Applications for Environmental Protection and Management. Energy, Environment, and Sustainability.

[11] Nikolaivits E., Agrafiotis A., Termentzi A., Machera K., Le Goff G., Álvarez P., Chavanich S., Benayahu Y., Ouazzani J., Fokialakis N. (2019). Unraveling the detoxification mechanism of 2,4-dichlorophenol by marine-derived mesophotic symbiotic fungi isolated from marine invertebrates. Mar. Drugs.

[12] Vroumsia T., Steiman R., Seigle-Murandi F., Benoit-Guyod J.-L. (2005). Groupe pour l’Étude du Devenir des Xénobiotiques dans l’Environnement (GEDEXE) Fungal bioconversion of 2,4-dichlorophenoxyacetic acid (2,4-D) and 2,4-dichlorophenol (2,4-DCP). Chemosphere.

[13] Matafonova G., Shirapova G., Zimmer C., Giffhorn F., Batoev V., Kohring G.-W. (2006). Degradation of 2,4-dichlorophenol by *Bacillus* sp. isolated from an aeration pond in the Baikalsk pulp and paper mill (Russia). Int. Biodeterior. Biodegrad..

[14] Kargi F., Eker S. (2005). Kinetics of 2,4-dichlorophenol degradation by *Pseudomonas putida* CP1 in batch culture. Int. Biodeterior. Biodegrad..

[15] Chen A., Zeng G., Chen G., Fan J., Zou Z., Li H., Hu X., Long F. (2011). Simultaneous cadmium removal and 2,4-dichlorophenol degradation from aqueous solutions by *Phanerochaete chrysosporium*. Appl. Microbiol. Biotechnol..

[16] Stoilova I., Krastanov A., Stanchev V., Daniel D., Gerginova M., Alexieva Z. (2006). Biodegradation of high amounts of phenol, catechol, 2,4-dichlorophenol and 2,6-dimethoxyphenol by *Aspergillus awamori* cells. Enzyme Microb. Technol..

[17] Subbotina N.M., Kolomytseva M.P., Baskunov B.P., Golovlev L.A. (2016). Catechol 1,2-dioxygenase induced in *Rhodococcus opacus* strain 1CP cultured in the presence of 3-hydroxybenzoate. Microbiology.

[18] Lin J., Milase R.N. (2015). Purification and characterization of catechol 1,2-dioxygenase from *Acinetobacter* sp. Y64 strain and *Escherichia coli* transformants. Protein J..

[19] Guzik U., Hupert-Kocurek K., Sitnik M., Wojcieszyńska D. (2013). High activity catechol 1,2-dioxygenase from Stenotrophomonas maltophilia strain KB2 as a useful tool in cis,cis-muconic acid production. Antonie Leeuwenhoek Int. J. Gen. Mol. Microbiol..

[20] Giedraityte G., Kalėdienė L. (2009). Catechol 1,2-dioxygenase from α-naphthol degrading thermophilic *Geobacillus* sp. strain: Purification and properties. Open Life Sci..

[21] Santos V.L., Linardi V.R. (2004). Biodegradation of phenol by a filamentous fungi isolated from industrial effluents-identification and degradation potential. Process Biochem..

[22] Cai W., Li J., Zhang Z. (2007). The characteristics and mechanisms of phenol biodegradation by *Fusarium* sp.. J. Hazard. Mater..

[23] Tsai S.-C., Li Y.-K. (2007). Purification and characterization of a catechol 1,2-dioxygenase from a phenol degrading *Candida albicans* TL3. Arch. Microbiol..

[24] Long Y., Yang S., Xie Z., Cheng L. (2016). Cloning, expression, and characterization of catechol 1,2-dioxygenase from a phenol-degrading *Candida tropicalis* JH8 strain. Prep. Biochem. Biotechnol..

[25] El-Naas M.H., Mousa H.A., Gamal M., Singh S.N. (2017). El Microbial degradation of chlorophenols. Microbe-Induced Degradation of Pesticides.

[26] Marco-Urrea E., Reddy C.A., Singh S. (2012). Degradation of chloro-organic pollutants by white rot fungi. Microbial Degradation of Xenobiotics. Environmental Science and Engineering.

[27] Aranda E. (2016). Promising approaches towards biotransformation of polycyclic aromatic hydrocarbons with Ascomycota fungi. Curr. Opin. Biotechnol..

[28] Nakagawa A., Osawa S., Hirata T., Yamagishi Y., Hosoda J., Horikoshi T. (2006). 2,4-Dichlorophenol degradation by the soil fungus *Mortierella* sp.. Biosci. Biotechnol. Biochem..

[29] Arora P., Bae H. (2014). Bacterial degradation of chlorophenols and their derivatives. Microb. Cell Fact..

[30] Rubilar O., Diez M.C., Gianfreda L. (2008). Transformation of chlorinated phenolic compounds by white rot fungi. Crit. Rev. Environ. Sci. Technol..

[31] Hupert-Kocurek K., Wojcieszyſska D., Guzik U., Borowski T., Wojcieszyńska D., Guzik U., Borowski T. (2015). A single amino acid substitution within catalytically non-active N-terminal domain of catechol 2,3-dioxygenase (C23O) increases enzyme activity towards 4-chlorocatechol. J. Mol. Catal. B Enzym..

[32] Gioti A., Siaperas R., Nikolaivits E., Le Goff G., Ouazzani J., Kotoulas G., Topakas E. (2020). Draft genome sequence of a *Cladosporium* species isolated from the mesophotic ascidian *Didemnum maculosum*. Microbiol. Resour. Announc..

